# Supplementary material for: Characterization of congenital hyperinsulinism in Argentina: Clinical features, genetic findings, and treatment outcomes
Source: PLoS One. 2025 Aug 19;20(8):e0321244. doi: 10.1371/journal.pone.0321244 (PMC12364331; doi:10.1371/journal.pone.0321244)
Supplement: Attachment — Submitted filename: 20250501 PLOS CHI Comments to the Author.docx [file pone.0321244.s001.docx]

**Comments to the Author**

The authors summarize genetic analyses of congenital hyperinsulinism (CHI) in Argentina. Given the paucity of reports on CHI-related genetic analyses from South America, this study represents a valuable contribution. The manuscript is generally well-written and presented. However, the reviewers have several questions that should be addressed prior to publication.

**Major Comments**

**P5L129**: Did the authors examine the intronic regions of the HK1 gene in all cases that were negative for known pathogenic variants?

**P7L174**: Did the individuals with late-onset CHI carry any pathogenic variants? Additionally, how was the diagnosis made in the third individual?

**P7L178**: The reported diagnostic yield appears lower than in other studies. Could the authors clarify the potential reasons for this discrepancy? For instance, is the allele frequency of pathogenic variants lower in this population than in other ethnic groups?

**P7L180–181**: Reference 36 mentions the 20p11.2 deletion syndrome, yet the current manuscript provides limited molecular genetic details. Similarly, could the authors elaborate further on the preoperative management for the patient with Turner syndrome? Was octreotide trialed? These details would enhance the clarity and clinical relevance of the manuscript.

**P10L278**: The authors state that access to genetic testing is more limited than 18F-DOPA PET-CT. However, in terms of actual cost, genetic testing is generally considered less expensive. As noted in P5L121, genetic testing was commercially conducted in six patients. Could the authors explain more about access issues in Argentina, such as insurance coverage or national policy implications?

**P13–14, Table 1 (Cases 8, 19, 21)**: The authors state that three patients with diffuse disease underwent surgery, yet the current treatment includes diazoxide in two patients and no treatment in one. Does this imply that diazoxide was effective even postoperatively? A summary of both preoperative and postoperative treatments would improve clarity.

**P14, Table 1 (Case 16)**: Was the use of alpha-glucosidase inhibitors considered for the patient with an INSR variant?

**Minor Comments**

**P8L205**: Were the three patients with diffuse disease genetically confirmed cases? If so, providing this information would improve clarity.

**P8L218**: Please correct the formatting issue—this text portion appears to be unnecessarily italicized.

**P13, Table 1**: It would be more intuitive to arrange the variants in order of amino acid position (e.g., p.Arg168His before p.Arg598Ter) to facilitate a more straightforward interpretation.

**P13, Table 1**: Please indicate whether there are any late-onset CHI cases in the table.
